# Osteoarticular tuberculosis cases in the southwest of China: A 9-year retrospective study

**DOI:** 10.3389/fmed.2023.1051620

**Published:** 2023-02-07

**Authors:** Dong-Mei Wang, Qi An, Qing Yang, Yi Liao, Yao Jian

**Affiliations:** ^1^Department of Science and Education Division, Public Health Clinical Center of Chengdu, Chengdu, Sichuan, China; ^2^Department of Clinical Laboratory Medicine, School of Medicine, Chengdu Women's and Children's Central Hospital, University of Electronic Science and Technology of China, Chengdu, China; ^3^Quality Management Department, Public Health Clinical Center of Chengdu, Chengdu, Sichuan, China

**Keywords:** osteoarticular, tuberculosis, epidemiology, clinical characteristics, drug resistance

## Abstract

**Background:**

Osteoarticular tuberculosis (TB) is an uncommon form of extrapulmonary TB. In this study, we analyzed the epidemiological characteristics, common sites, and drug resistance profiles of osteoarticular TB infections occurring in southwest China.

**Methods:**

A total of 3,254 cases of patients clinically diagnosed with osteoarticular TB infections between 2013 and 2021 were retrospectively analyzed. Patients' demographic and clinical characteristics were collected. Drug sensitivity testing was performed using the microporous plate ratio method. Chi-squared analysis was used to analyze the rates of and trends in mycobacterial isolates.

**Results:**

Of the 3,254 patients, 1,968 (60.5%) were men and boys, and 1,286 (39.5%) were women and girls; patients' ages ranged from 1 to 91 years, with an average of 42 ± 19.3 years. In terms of disease, 2,261 (69.5%) had spinal TB, mainly thoracic (815, 36%) or lumbar (1,080, 48%); joint TB was found in 874 cases (26.9%), mainly occurring in the knee (263, 30%) or hip (227, 26%); and both spinal and joint TB were observed in 119 cases (3.7%). Drug susceptibility tests were performed on 241 isolated strains of MTB; 70 strains (29.0%) were resistant to at least one drug, and MDR-TB and XDR-TB were observed in 7.1 and 1.2% of strains, respectively.

**Conclusions:**

In southwest China over this period, osteoarticular TB mainly affected middle-aged and young men with poor nutritional status. Patients from ethnic minority areas also accounted for a large proportion of cases. Spinal TB is prone to occur in the lumbar and thoracic vertebrae, and joint TB is prone to occur in the lower limb joints. Additionally, there has been an increasing trend in the number of TB cases over the past 9 years, and drug resistance has also increased.

## Introduction

Tuberculosis (TB) is a chronic infectious disease that seriously endangers human health. It can affect almost all organs and tissues of the body, but most commonly occurs in the lungs (80% of cases). Other infection sites may include the lymph nodes, bone joints, meninges, etc. In terms of extra-pulmonary tuberculosis (EPTB), cases involving the osteoarticular system account for about 10% of all cases (1). The cure rate for osteoarticular TB is poor, especially considering the high rates of drug-resistant TB and of complications, which may lead to serious consequences (2–5). Therefore, exploring the regional epidemiology of this form of the disease and its clinical characteristics, common infection sites, bacterial types, and drug resistance is of crucial importance for the diagnosis and treatment of various types of osteoarticular TB.

This study retrospectively analyzed demographic data relating to 3,254 cases of osteoarticular TB infection clinically diagnosed in a central city hospital in southwest China between January 2013 and December 2021; the epidemiological characteristics, common infection sites, bacterial types, and drug resistance of these cases of osteoarticular TB infection were comprehensively analyzed in order to provide data to support the clinical diagnosis and treatment of osteoarticular TB.

## Methods

### Study population and diagnostic criteria

The present study included medical records of all patients with osteoarticular TB infection who were identified in the medical records system of Chengdu PHCC in Sichuan Province, China, as having been treated there between January 2013 and December 2021. The inclusion criteria were as follows: the WHO guidelines for TB diagnosis (5) and the clinical diagnosis standard for TB issued by the Chinese Medical Association (6) were met; screening imaging showed typical osteoarticular TB infection; *Mycobacterium* TB (MTB) was isolated and cultured from bone or pus from the affected site, or acid-fast smear of tissue samples was positive; and the pathology of the specimen showed caseous necrosis and granulomatous inflammation. Cases of disease confirmed to be osteoarticular *Mycobacterium* TB infection by various other means were excluded.

During the 9-year period of the study, suspected osteoarticular TB infections were cultured using the BACTEC™ MGIT 960 system (Becton Dickinson & Co., NJ, USA). Strains of the same mycobacteria cultured multiple times from the same patient and the same site were not counted repeatedly. The medical records of all selected participants, including epidemiology, clinical features, and laboratory test results, were retained.

This study was approved by the Ethics Committee of the Public Health Clinical Center (PHCC) of Chengdu. All patient information used in this study was routinely collected through the mandatory notification system. The ethics committee waived the requirement for informed consent.

### Culture, identification, and drug sensitivity testing of bacterial strains

The BACTEC™ MGIT 960 system was used for mycobacteria culture. Pathological specimens, pus, and caseous materials from bone lesions were collected by puncture or surgery, as previously reported (7). MPT 64 antigen detection (colloidal gold immunochromatography), P-nitrobenzoic acid (PNB), and thiophene-2-carboxylic acid hydrazide (TCH) were used for identification of MTB/NTM (8), while polymerase chain reaction (PCR) was used for further identification of the species/complex, in accordance with the manufacturer's instructions (CapitalBio Corp., Chengdu, China) (9).

Drug-susceptibility testing (DST) of the culture-positive MTB isolates was performed using MicroDST^TM^ (Yinke AUTOBIO Diagnostics Co., Ltd., Zhuhai, China). The minimum inhibitory concentration (MIC) was defined as the lowest concentration of the drug that inhibited visible growth of the tested isolates. MIC breakpoints, sensitivity, and resistance were determined in accordance with the reagent instructions, and the protocol was executed in accordance with the manufacturer's recommendations (7). A total of 14 antimicrobial agents were utilized in this study, namely isoniazid (INH, 0.4–1.6 μg/mL), rifampicin (RIF, 2–8 μg/mL), ethambutol (EMB, 5–20 μg/mL), streptomycin (STR, 2–8 μg/mL), levofloxacin (LFX, 2–8 μg/mL), amikacin (AMK, 1–4 μg/mL), capreomycin (CM, 2.5–10 μg/mL), moxifloxacin (MOX, 0.5–2 μg/mL), rifabutin (RFB, 0.75–3 μg/mL), protionamide (PTO, 10–40 μg/mL), dipasic (DIP, 0.5–2 μg/mL), kanamycin (KM, 2.5–10 μg/mL), clofazimine (CFZ, 2–8 μg/mL), and paza-aminosalicylatel (PAS, 2–8 μg/mL) at four MICs. In addition, the control strain H37Rv was monitored.

### Laboratory quality control

External quality assessment (EQA) was conducted on the smear, culture, and DST procedures at the Innovation Alliance on Tuberculosis Diagnosis and Treatment (Beijing, China). Specifically, blinded retesting of a random selection of ≈10% of isolates from the study laboratory was conducted at a superior laboratory.

### Statistical analysis

Data were analyzed using the SPSS Statistics client 19.0 (SPSS Inc., IL, USA). Normally distributed measurement data are reported in the form of means, and categorical variables are reported in the form of number and percentage. Chi-squared analysis was used to analyze the rates of and trends in mycobacterial isolates over the course of 9 years. The threshold for statistical significance was set at *P* < 0.05.

## Results

### Demographic and clinical characteristics

**Figure 1:**
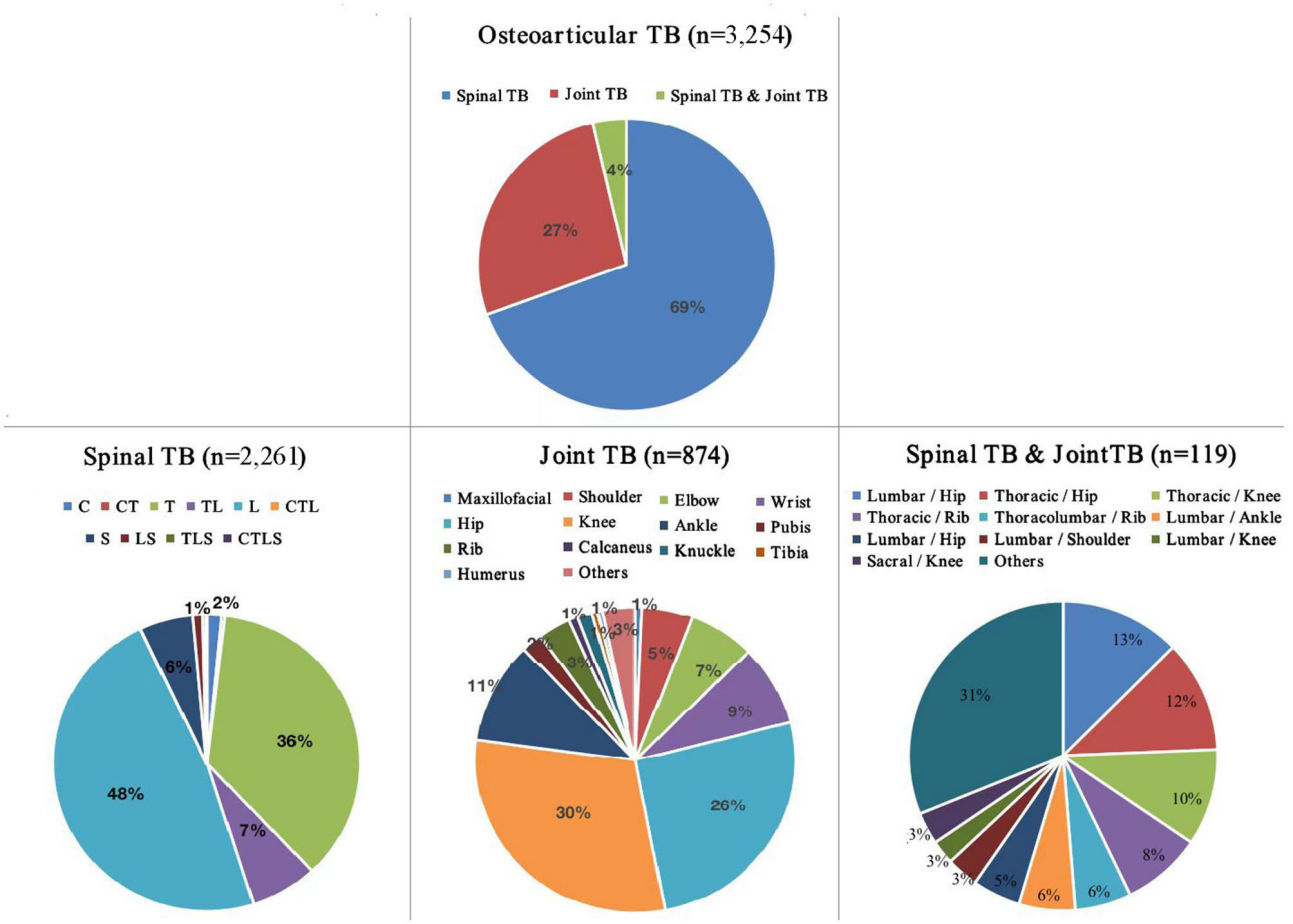
Distribution of sites of osteoarticular tuberculosis infection among patients in southwest China between 2013 and 2021. TB, tuberculosis; C, cervical; T, thoracic; L, lumbar; S, sacral; *n* = 3,254.

**Figure 2:**
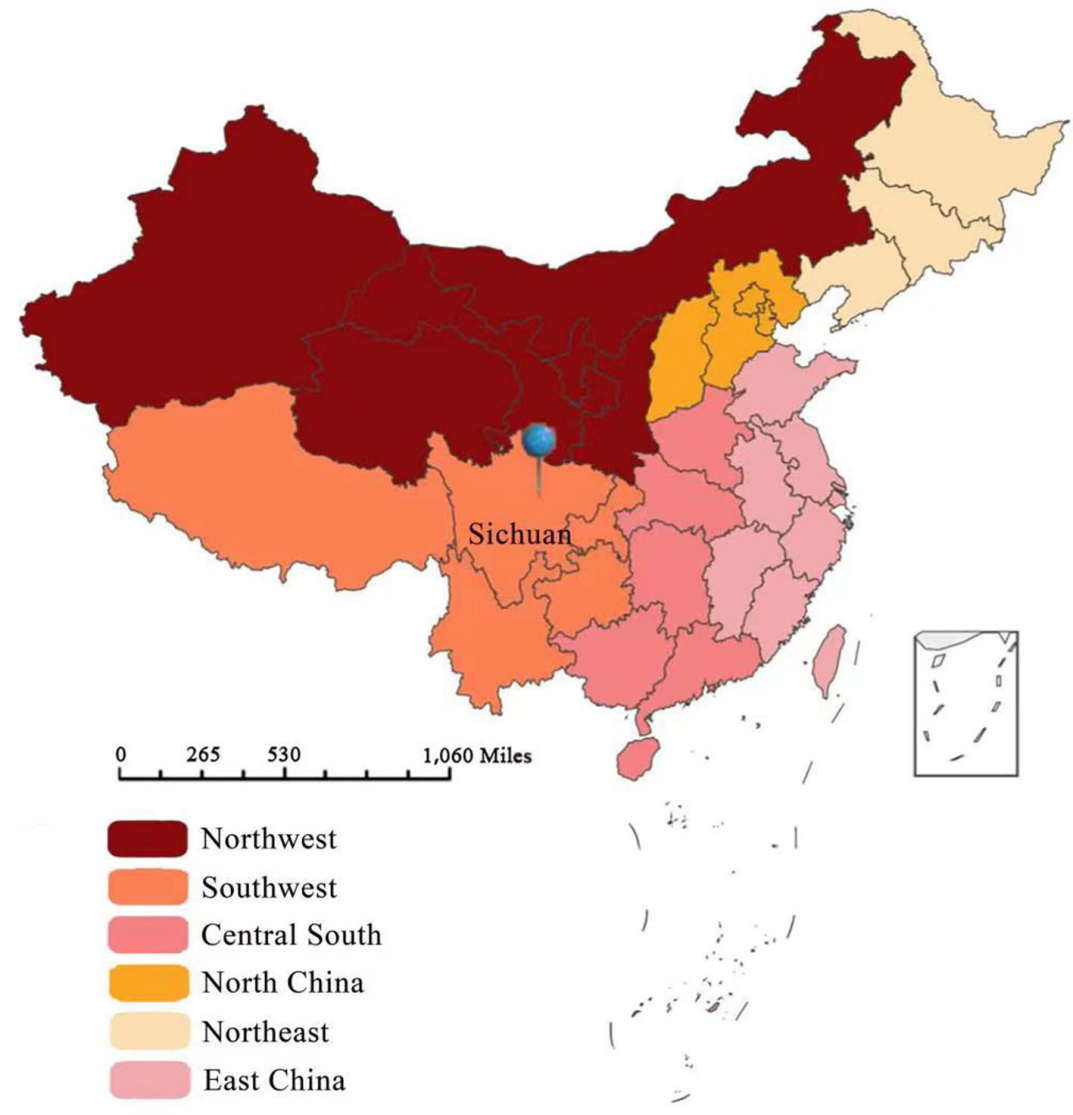
Geographical location of research center (blue pin).

Specimens from suspected cases were cultured for mycobacteria using the BACTEC™ MGIT 960 system between January 2013 and December 2021. A total of 3,254 patients with clinically diagnosed osteoarticular *Mycobacterium* TB infection were treated at PHCC (Figures 1, 2). The mean age of these 3,254 osteoarticular TB patients was 42 + 19.3 years (range: 1–91); the sample comprised 60.5% men and boys (*n* = 1,970) and 39.5% women and girls (*n* = 1,289), with a male:female ratio of 1.5:1. The quartiles for age were 27, 42, and 57 years; only 246 patients (7.6%) were < 18 years old. Patients aged 18–39 years accounted for 38.8% of the sample (*n* = 1,262); those aged 40–60 years accounted for 31.78% (*n* = 1,034); and those aged >60 years accounted for 21.9% of the cohort (*n* = 712), as shown in Table 1. In addition, 1,298 of the patients (39.9%) belonged to ethnic minorities. Furthermore, 453 patients (13.9%) had at least one co-morbid disease: pulmonary infection occurred in 872 patients (26.8%), hypoproteinemia in 763 (23.4%), anemia in 736 (22.6%), hypertension in 375 (11.5%), malnutrition in 314 (9.6 %), diabetes mellitus in 271 (8.3%), hepatitis B virus in 219 (6.7%), cerebral infarction in 194 (6.0 %), HIV infection in 170 (5.2%), heart disease in 100 (3.1%), thyroid dysfunction in 55 (1.7%), malignancy in 65 (2.0%), syphilis in 49 (1.5%), renal failure in 42 (1.3%), epilepsy in 39 (1.2%), hepatitis C in 35 (1.1%), and diseases of the immune system in 34 (1.0%). A number of patients experienced at least one adverse drug reaction: hyperuricemia occurred in 1,067 patients (32.8%), liver dysfunction in 801 (24.6%), leukopenia in 223 (6.9%), thrombocytopenia in 95 (2.9 %), and rash in 63 (1.9%).

**Table 1:** General characteristics of patients with osteoarticular tuberculosis infection (*n* = 3,254).

**Variable**	**Total (%)**
**Sex**	
Female	1,286 (39.52)
Male	1,968 (60.48)
**Age**	
Mean ± SD; years (range)	42 + 19.3 (1–91)
< 18 (male, female)	246 (144, 102) (7.56)
18–39 (male, female)	1,262 (769, 493) (38.78)
40–60 (male, female)	1,034 (646, 388) (31.78)
>60 (male, female)	712 (409, 303) (21.88)
**Ethnicity**	
Han	1,956 (60.11)
Tibetan	1,143 (35.13)
Yi	132 (4.06)
Qiang	13 (0.40)
Others	10 (0.31)
Concomitant PTB	2,074 (63.74)
Concomitant tuberculous meningitis	148 (4.55)
Concomitant tuberculous pleurisy	960 (29.50)
Concomitant tuberculous lymphadenitis	582 (17.89)
Concomitant peritoneal tuberculosis	7 (0.22)
**Two or more sites of concomitant osteoarticular TB**	
Bone destruction	71 (2.18)
Sinus tract	106 (3.26)
Paraplegia	124 (3.81)
**Co-morbid infectious disease**	
AIDS	170 (5.22)
Diabetes	271 (8.33)
Anemia	736 (22.62)
Hepatitis B	219 (6.73)
Hepatitis C	35 (1.08)
Hypertension	375 (11.52)
Maligancy	65 (2.00)
Heart disease	100 (3.07)
Diseases of the immune system	34 (1.04)
Pulmonary infection	872 (26.80)
Hypoproteinemia	763 (23.45)
Malnutrition	314 (9.65)
Syphilis	49 (1.51)
Cerebral infarction	194 (5.96)
Epilepsy	39 (1.20)
Thyroid dysfunction	55 (1.69)
Renal failure	42 (1.29)
**Adverse drug reaction**	
Liver dysfunction	801 (24.62)
Rash	63 (1.94)
Hyperuricemia	1,067 (32.79)
Leukopenia	223 (6.85)
Thrombocytopenia	95 (2.92)

TB, tuberculosis; PTB, pulmonary tuberculosis.

### Site distribution in cases of osteoarticular TB infection

Among the 3,254 cases of osteoarticular TB infection, 2,261 (69.5%) were cases of spinal TB. The sites of spinal TB were divided into the cervical vertebrae (1–6), cervicothoracic region (cervical 7-thoracic 2), thoracic vertebrae (3–11), thoracolumbar region (thoracic 12, lumbar 1), lumbar vertebrae (2–4), lumbosacral region (lumbar 5, sacral 1), sacral vertebra, and coccyx. The results of statistical analysis showed that the lesions involved in spinal TB cases occurred in the lumbar (48%), thoracic (36%), sacral (6%), and cervical (2%) regions.

There were 874 cases of joint TB (26.9% of the total cases), most of which occurred in the knee (30%), hip (26%), or ankle (11%); other joint TB sites were relatively rare. One hundred nineteen cases (3.7% of the total) involved spinal TB and joint TB occurring at multiple sites; these cases mainly involved sites in the thoracolumbar segment along with lower limb joints, and the upper limb joints were relatively rarely involved (Figure 1).

### Drug resistance of the MTB species

**Figure 3:**
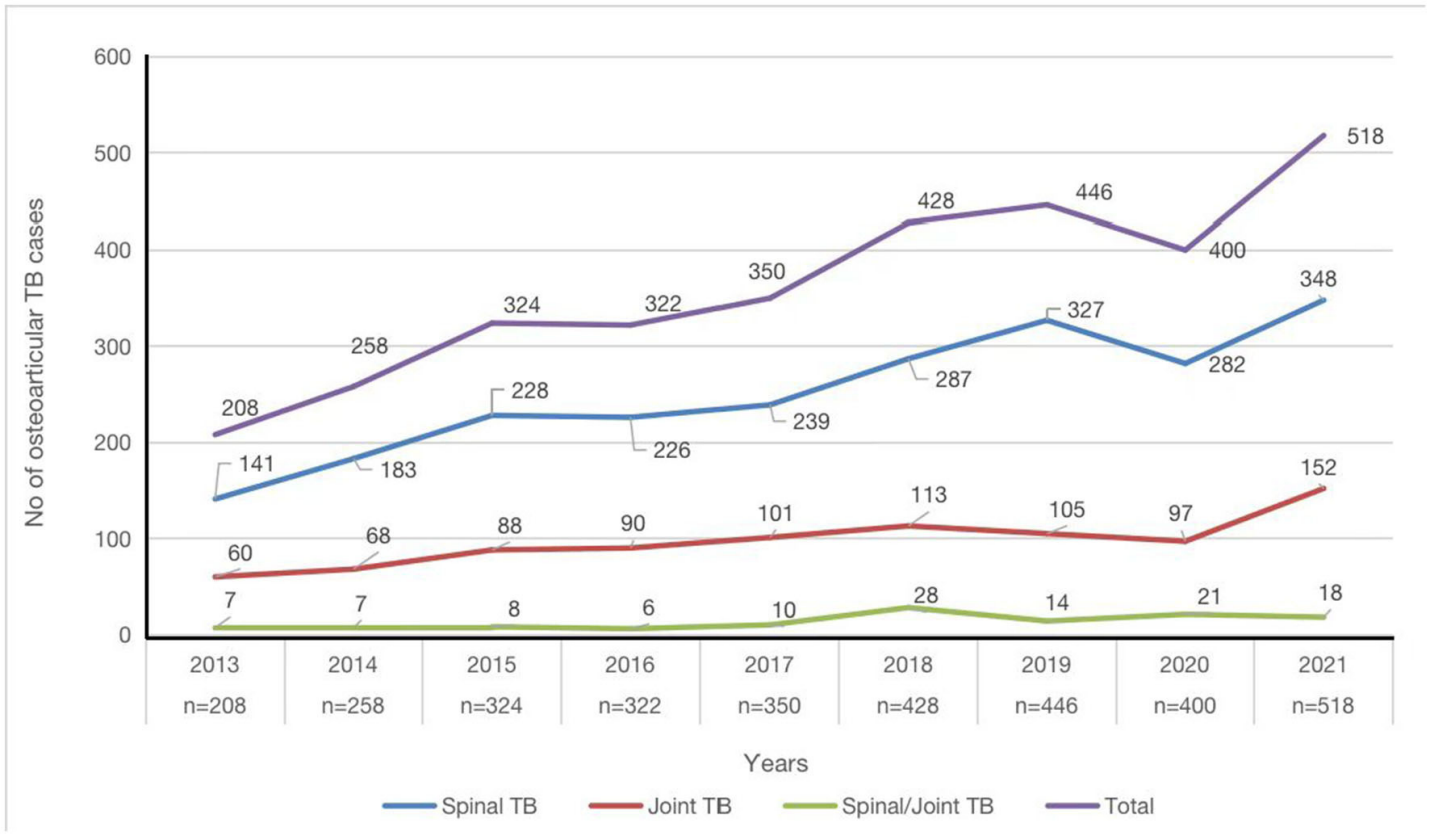
Numbers of patients with various types of osteoarticular tuberculosis infection presenting each year between 2013 and 2021 (*n* = 3,254).

DST of 241 cases of osteoarticular TB with positive MTB culture *in vitro* indicated resistance to any ATD in the case of 70 isolates (29.0%), with 45 cases of first-line drug resistance (18.7%) and 51 cases of second-line drug resistance (21.2%). In addition, 17 isolates (7.1%) and 3 isolates (1.2%) were identified as MDR-TB and XDR-TB, respectively. The numbers of the 241 cases exhibiting resistance to individual ATDs (from highest to lowest) were as follows: INH (high-level resistance >1.6 μg/ml): 55 cases (22.8%); RIF (high-level resistance > 8.0 μg/ml): 37 cases (15.4%); DIP (high-level resistance > 2.0 μg/ml): 30 cases (12.4%); STR (high-level resistance > 8.0 μg/ml): 27 cases (11.2%); PTO (high-level resistance > 40.0 μg/ml): 27 cases (11.2%); RFB (high-level resistance > 3.0 μg/ml): 25 cases (10.4%); EMB (high-level resistance > 20.0 μg/ml): 11 cases (4.6%); LFX (high-level resistance > 8.0 μg/ml), MOX (high-level resistance > 2.0 μg/ml), and PAS (high-level resistance > 20.0 μg/ml): 6 cases (2.5%) each; CM (high-level resistance > 10.0 μg/ml): 4 cases (1.7%); AMK (high-level resistance > 4.0 μg/ml): 3 cases (1.2%); KM (high-level resistance > 10.0 μg/ml) and CFZ (high-level resistance > 8.0 μg/ml): 1 case (0.4%) each. In addition, isolates from 5 of the 241 samples (2.1%) were resistant to isoniazid and streptomycin; 8 (3.3%) to INH, RIF, and STR; 3 (1.2%) to INH, RIF, and EMB; and 1 (0.4%) to INH, RIF, STR, and EMB (Table 2). The incidence of *Mycobacterium* osteoarticular infection increased year on year (*P* < 0.05) (spinal TB > joint TB > spinal TB and joint TB) (Figure 3).

**Table 2:** Drug resistance of clinical isolates from patients with different types of osteoarticular tuberculosis (*n* = 241).

**Drug species**	**Spinal TB (*****n*** = **141)**							**Joint TB (*****n*** = **95)**										**Spinal TB and joint TB (*****n*** = **5)**						**Total (%) (*n* = 241)**
	**C (*n* = 4)**	**T (*n* = 49)**	**TL (*n* = 16)**	**L (*n* = 59)**	**LS (*n* = 8)**	**S (*n* = 5)**	**Spinal total (%)**	**M (*n* = 2)**	**SH (*n* = 5)**	**E (*n* = 7)**	**W (*n* = 12)**	**H (*n* = 22)**	**K (*n* = 22)**	**A (*n* = 20)**	**O # (*n* = 3) **	**P (*n* = 2)**	**Joint total (%)**	**T/K (*n* = 1)**	**L/K (*n* = 1) **	**L/P (*n* = 1)**	**T/W (*n* = 1) **	**L/H (*n* = 1)**	**Spinal and joint total (%)**	
Any drug resistance^*^	0	16	7	15	4	2	44 (31.2)	1	2	0	5	3	10	4	1	0	26 (27.4)	0	0	0	0	0	0 (0.0)	70 (29.0)
Any first-line drug resistance	0	6	4	10	4	1	25 (17.7)	1	2	0	3	2	8	3	1	0	20 (21.1)	0	0	0	0	0	0 (0.0)	45 (18.7)
Any second-line drug resistance	0	14	6	11	3	1	35 (24.8)	1	2	0	3	1	6	3	0	0	16 (16.8)	0	0	0	0	0	0 (0.0)	51 (21.2)
INH	2	8	6	15	3	1	35 (24.8)	1	2	0	2	5	7	1	0	0	18 (18.9)	1		1		0	2 (40.0)	55 (22.8)
RIF	1	4	3	12	1	1	22 (15.6)	0	1	1	2	3	6	2	0	1	15 (15.8)	0	0	0	0	0	0 (0.0)	37 (15.4)
EMB	1	3	1	5	0	0	10 (7.1)	0	0	0	0	0	1	0	0	0	1 (1.1)	0	0	0	0	0	0 (0.0)	11 (4.6)
STR	1	5	2	9	4	0	21 (14.9)	1	0	0	2	0	1	1	1	0	6 (6.3)	0	0	0	0	0	0 (0.0)	27 (11.2)
LFX	1	1	1	0	1	0	4 (2.8)	0	0	0	1	1	0	0	0	0	2 (2.1)	0	0	0	0	0	0 (0.0)	6 (2.5)
AMK	1	0	0	0	0	0	1 (0.7)	0	1	0	1	0	0	0	0	0	2 (2.1)	0	0	0	0	0	0 (0.0)	3 (1.2)
CM	1	1	0	1	0	0	3 (2.1)	0	1	0	0	0	0	0	0	0	1 (1.1)	0	0	0	0	0	0 (0.0)	4 (1.7)
MOX	1	1	1	0	1	0	4 (2.8)	0	1	0	1	0	0	0	0	0	2 (2.1)	0	0	0	0	0	0 (0.0)	6 (2.5)
RFB	1	5	2	9	0	1	18 (12.8)	0	0	0	3	0	2	2	0	0	7 (7.4)	0	0	0	0	0	0 (0.0)	25 (10.4)
PTO	1	10	4	4	1	0	20 (14.2)	1	1	0	0	0	4	0	0	0	6 (6.3)	1	0	0	0	0	1 (20.0)	27 (11.2)
DIP	1	6	1	13	1	0	22 (15.6)	1	0	0	1	0	3	2	0	0	7 (7.4)	1	0	0	0	0	1 (20.0)	30 (12.4)
KM	1	0	0	0	0	0	1 (0.7)	0	0	0	0	0	0	0	0	0	0 (0.0)	0	0	0	0	0	0 (0.0)	1 (0.4)
CFZ	1	0	0	0	0	0	1 (0.7)	0	0	0	0	0	0	0	0	0	0 (0.0)	0	0	0	0	0	0 (0.0)	1 (0.4)
PAS	1	0	1	3	0	0	5 (3.6)	0	0	0	1	0	0	0	0	0	1 (1.1)	0	0	0	0	0	0 (0.0)	6 (2.5)
MDR (INH+RIF)	0	2	2	6	1	1	12 (8.5)	0	0	0	0	0	4	1	0	0	5 (5.3)	0	0	0	0	0	0 (0.0)	17 (7.1)
Pre-XDR	0	1	1	0	0	0	2 (1.4)	0	0	0	0	0	0	0	0	0	0 (0.0)	0	0	0	0	0	0 (0.0)	2 (0.8)
XDR	1	0	0	0	0	0	1 (0.7)	0	1	0	1	0	0	0	0	0	2 (2.1)	0	0	0	0	0	0 (0.0)	3 (1.2)
INH+STR	0	1	0	0	2	0	3 (2.1)	1	0	0	0	0	1	0	0	0	2 (2.1)	0	0	0	0	0	0 (0.0)	5 (2.1)
INH+RIF+STR	0	1	1	4	0	0	6 (4.3)	0	0	0	1	0	1	0	0	0	2 (2.1)	0	0	0	0	0	0 (0.0)	8 (3.3)
INH+RIF+EMB	0	2	1	0	0	0	3 (2.1)	0	0	0	0	0	0	0	0	0	0 (0.0)	0	0	0	0	0	0 (0.0)	3 (1.2)
RIF+STR+EMB	0	0	0	0	0	0	0 (0.0)	0	0	0	0	0	0	0	0	0	0 (0.0)	0	0	0	0	0	0 (0.0)	0 (0.0)
INH+RIF+STR +EMB	0	0	0	1	0	0	1 (0.7)	0	0	0	0	0	0	0	0	0	0 (0.0)	0	0	0	0	0	0 (0.0)	1 (0.4)

TB, tuberculosis; C, cervical; T, thoracic; TL, thoracolumbar; L, lumbar; LS, lumbosacral; S, sacrococcygeal; M, maxillofacial; SH, shoulder; E, elbow; W, wrist; H, hip; K, knee; A, ankle; O, others; T/A, thoracolumbar/ankle; L/K, lumbosacral/knee; L/P, lumbar/pubic; T/W, thoracic/wrist.

^*^Resistant to at least one drug.

^#^Others include pubis (1), navicular (1), and humerus (1). INH, isoniazid; STR, streptomycin; RIF, rifampicin; EMB, ethambutol; LFX, levofloxacin; AMK, amikacin; CM, capreomycin; MOX, moxifloxacin; RFB, rifabutin; PTO, protionamide; DIP, dipasic; KM, kanamycin; CFZ, clofazimine; PAS, paza-aminosalicylatel; MDR-TB, multidrug-resistant tuberculosis; XDR, extensively drug-resistant tuberculosis.

### Treatment and outcomes

The basic anti-TB treatment plan for the 3,254 patients with osteoarticular TB was 6HREZ/12HRE. However, this anti-TB treatment program was strengthened according to the clinical situation of preoperative and postoperative patients. Additionally, when evaluation of the efficacy of the anti-TB treatment plan indicated poor efficacy or a definite diagnosis of drug resistance was made, the treatment regimen could be adjusted. Among all the cases studied, 35 patients died, 59 did not recover, 50 were cured, and 3,110 improved.

## Discussion

Osteoarticular TB is an uncommon type of TB that causes significant functional impairment. With the development of the disease, bone joint and vertebral bone destruction further lead to joint deformity and even disability. Caseous necrosis, nodule formation, and proliferation of granulation tissue are often seen in patients with osteoarticular TB. The positive rate of molecular diagnosis or clinical pathogenic detection *via* smear testing of such tissues or joint fluid is low. Furthermore, the positive rate of bacterial culture is lower still, due to the long-term use of anti-TB drugs and factors such as material selection and culture. Therefore, clinical diagnosis and treatment rely on empirical drugs (10–13). Above, we have summarized and analyzed the epidemiological characteristics, common infection sites, bacterial types, and drug resistance profiles of 3,254 clinically confirmed cases of osteoarticular TB infection occurring at Chengdu PHCC in southwest China between January 2013 and December 2021; all of these characteristics are of particular significance for the diagnosis and treatment of various types of osteoarticular TB and for the clinical use of empirical drugs.

Osteoarticular TB more frequently affects young adults in countries with a high TB burden, while in developed countries, the disease is often seen in older individuals (14, 15). A previous study reported that female patients were more than twice as likely to have EPTB as compared to male patients (16). In contrast, in this study, we did not find an association between gender and osteoarticular TB disease. However, the male-to-female ratio in the sample of patients was 1.5:1, which is much lower than the ratio among Chinese PTB patients and that observed in previous research by our group on EPTB in southwest China (14, 17). In this study, osteoarticular TB more frequently affected young male adults (18–60 years).

Sichuan province is the gateway to the southwest of China, a multi-ethnic community with the second largest Tibetan region in China (9). In this study, most of the patients with osteoarticular TB who came from outside the province were from Tibet, Qinghai, or Ningxia, and most who came from within the province were from Gan Zi, Aba, Liang Shan, or Xi Chang, among other places. More than 40% of the patients were members of ethnic minorities. The aforementioned areas are economically underdeveloped areas with poor health conditions, where most of the residents are nomads. In these areas, the BCG vaccination rate is low, and residents' awareness of TB and its prevention and control is poor. Therefore, the government should further strengthen the prevention and control of TB in ethnic minority areas of western Sichuan and increase the coverage of the BCG vaccine.

Osteoarticular TB is often secondary to pulmonary tuberculosis, and is often accompanied by tuberculous pleurisy, meningitis, and TB at other sites. In this study, 63.6% of cases were complicated with pulmonary TB; some involved disseminated lesions, such as meningitis and pleural peritonitis to varying degrees, and 123 of the 3,254 cases (3.8%) involved simultaneously occurring osteoarticular TB lesions at two or more sites. The affected sites were mainly in the lumbar spine, followed by involvement of the thoracic vertebrae, cervical vertebrae, and sacral vertebra. The hip joint, knee, and sacroiliac joint were the most common sites in joint TB.

Among the cases of multiple-site combined incidence of spinal TB and joint TB, the most common sites were the thoracolumbar segment combined with lower limb joints, and involvement of the upper limb joints was relatively rare. We speculate that this may be related to the weight-bearing nature of the bone and joint: specifically, when the joint in question is weight-bearing to a significant degree, the incidence rate is high, and vice versa. At the same time, the fact that 736 (22.6%) and 763 (23.4%) of the 3,254 cases in this study were accompanied by anemia and hypoproteinemia, respectively, further suggests that pulmonary TB patients are more likely to develop secondary osteoarticular TB when their body is malnourished and their immunity is reduced. Therefore, throughout the entire cycle of TB treatment, we should strengthen nutritional support treatment, improve patients' immunity, and encourage patients to reduce the frequency of physical labor.

In this study, 241 MTB strains were isolated from 3,254 cases of osteoarticular TB, and *in vitro* drug sensitivity testing was carried out. The results showed that 45 MTB strains had varying degrees of resistance to INH, RIF, EMB, and STR. The rate of any first-line drug resistance was 18.7%, and the sequence of resistance rates was INH>RIF>STR>EMB. Additionally, 51 strains of MTB showed varying degrees of resistance to 10 second-line anti-TB drugs, such as LFX, AMK, or CM. The rate of any second-line drug resistance was 21.2%, and the sequence of resistance rates was DIP>PTO>RFB>LFX=PAS=MOX>CM>AMK>KM=CFZ. The overall drug resistance rate was 29.0%, which can be regarded as very high. This could be because most of the patients in our hospital with osteoarticular TB infections were critically ill patients with complex diseases and severe forms of infection, and the rate of referrals was high. Therefore, the frequency of use of second-line drugs was relatively high. The rates of MDR and XDR were 7.1 and 1.2%, respectively.

In addition to the high rates of drug resistance, the total number of cases of osteoarticular TB infection has shown an upward trend over the past 9 years (*p* < 0.05). On the one hand, this may be related to improvements in people's awareness of disease prevention and control in recent years, meaning that the number of people seeking medical treatment has increased. On the other hand, it may be related to improvements in medical technology and clinical detection level, which have greatly improved the positive detection rate of pathogens.

## Conclusions

Osteoarticular TB patients in southwest China are mainly young and middle-aged men with poor nutritional status. Cases in ethnic minority areas of western Sichuan accounted for a large proportion of the total in this study. Spinal TB was mainly found in the thoracolumbar segment, and joint TB in the lower limb joints. The proportion of drug resistance was high, and the number of cases increased year-on-year over the past 9 years. Therefore, in the treatment of patients with osteoarticular TB, *Mycobacterium* culture and drug sensitivity testing should be carried out, drug resistance should be dynamically monitored, nutritional support treatment of patients should be strengthened, their immunity should be improved, and patients should reduce strenuous physical labor. Relevant government departments should further strengthen measures for the prevention and control of TB in ethnic minority areas of western Sichuan and spread awareness of osteoarticular TB.

## Data availability statement

The original contributions presented in the study are included in the article/supplementary material, further inquiries can be directed to the corresponding author.

## Ethics statement

This article was approved by the Ethics Committee of Public Health Clinical Center of Chengdu. Written informed consent to participate in this study was provided by the participants' legal guardian/next of kin. Written informed consent was obtained from the individual(s), and minor(s)' legal guardian/next of kin, for the publication of any potentially identifiable images or data included in this article.

## Author contributions

D-MW: conception and design, acquisition of data, and manuscript revision. QA, QY, and YJ: collection and assembly of data. YL: collection and assembly of data and manuscript writing. All authors: final approval of manuscript.

## References

[1] World Health Organization (WHO) Global Tuberculosis Report 2021. Geneva, Switzerland: WHO (2021). Available online at: https://www.who.int/publications/i/item/9789240037021 (accessed December 27, 2021).

[2] Migliori GB Tiberi S Zumla A Petersen E Chakaya JM Wejse C. MDR/XDR-TB management of patients and contacts: challenges facing the new decade. The 2020 clinical update by the global tuberculosis network. Int J Infect Dis. (2020) 92S:S15–25. 10.1016/j.ijid.2020.01.042 32032752

[3] Ugarte-Gil C Curisinche M Herrera-Flores E Hernandez H Rios J. Situation of the tuberculosis-diabetes comorbidity in adults in Peru: 2016–2018. Rev Peru Med Exp Salud Publica. (2021) 38:254–60. 10.17843/rpmesp.2021.382.6764 34468572

[4] Agudelo CA Álvarez MF Hidrón A Villa JP Echeverri-Toro LM Ocampo A. Outcomes and complications of hospitalised patients with HIV-TB co-infection. Trop Med Int Health. (2021) 26:82–8. 10.1111/tmi.13509 33155342

[5] *WHO: Tuberculosis (TB)*. (2020). Available online at: https://www.who.int/tb/areas-of-work/laboratory/en/

[6] Chinese Medical Association. Clinical Diagnosis Standard of TB for Clinical Technology Operation (TB Volumes). [in Chinese]. People's Medical Publishing House (2005).

[7] Wang DM Li QF Zhu M Xu YH Liao Y. Clinical characteristics, common sites and drug resistance profile in culture-confirmed extrapulmonary TB/HIV co-infection patients, Southwest China. J Glob Antimicrob Resist. (2022) 28:1–7. 10.1016/j.jgar.2021.10.028 34920176

[8] Cao XJ Li YP Wang JY Zhou J Guo XG. MPT64 assays for the rapid detection of *Mycobacterium tuberculosis*. BMC Infect Dis. (2021) 21:336. 10.1186/s12879-021-06022-w 33838648 PMC8035777

[9] Wang DM Li QF Zhu M Wu GH Li X Xu YH. Epidemiological, clinical characteristics and drug resistance situation of culture confirmed children TBM in southwest of China: a 6-year retrospective study. BMC Infect Dis. (2020) 20:318. 10.1186/s12879-020-05041-3 32357835 PMC7195785

[10] Zeng H Liang Y He J Chen L Su H Liao S. Analysis of clinical characteristics of 556 spinal tuberculosis patients in two tertiary teaching hospitals in Guangxi province. Biomed Res Int. (2021) 2021:1344496. 10.1155/2021/1344496 34926681 PMC8683179

[11] Liu Z Li W Zhang Y Wu Y Xiao X Sun Z. Analysis of clinical factors, bacterial genotyping, and drug resistance for spinal tuberculosis in south-central China. Biomed Res Int. (2020) 2020:9871390. 10.1155/2020/9871390 32076625 PMC6996694

[12] Khan A Singh R Sharma S Singh V Sheoran A Soni A. Diagnosis of osteoarticular tuberculosis by immuno-PCR assay based on mycobacterial antigen 85 complex detection. Lett Appl Microbiol. (2022) 74:17–26. 10.1111/lam.13567 34592012

[13] Jia H Pan L Qin S Liu F Du F Lan T. Evaluation of interferon-γ release assay in the diagnosis of osteoarticular tuberculosis. Diagn Microbiol Infect Dis. (2013) 76:309–13. 10.1016/j.diagmicrobio.2013.03.030 23647965

[14] Gunal S Yang Z Agarwal M Koroglu M Arici ZK Durmaz R. Demographic and microbial characteristics of extrapulmonary tuberculosis cases diagnosed in Malatya, Turkey, 2001-2007. BMC Public Health. (2011) 11:154. 10.1186/1471-2458-11-154 21385458 PMC3060117

[15] Al-Hajoj S Shoukri M Memish Z AlHakeem R AlRabiah F Varghese B. Exploring the sociodemographic and clinical features of extrapulmonary tuberculosis in Saudi Arabia. PLoS ONE. (2015) 10:e0101667. 10.1371/journal.pone.0101667 25647300 PMC4315397

[16] Ong A Creasman J Hopewell PC Gonzalez LC Wong M Jasmer RM. Molecular epidemiological assessment of extrapulmonary tuberculosis in San Francisco. Clin Infect Dis. (2004) 38:25–31. 10.1086/380448 14679444

[17] Wang DM Li QF Zhu M Xu YH Luo J Li YJ. Analysis of infection and drug-resistance in 6 107 cases of extrapulmonary tuberculosis in Chengdu area [in Chinese]. Zhonghua Jie He He Hu Xi Za Zhi. (2017) 40:592–5. 10.3760/cma.j.issn.1001-0939.2017.08.010 28810312

